# COMPARISON OF DIAGNOSTIC COMPLEXITY AND IMAGING DECISIONS IN YOUNGER, MIDDLE AGE, AND OLDER ADULTS

**DOI:** 10.1093/geroni/igad104.2980

**Published:** 2023-12-21

**Authors:** Varun Saraswathula, Aubrey Mwinyogle, Lili Schadt, Gopal Kowdley, Rachel Kelz, Ari Friedman

**Affiliations:** University of Pennsylvania, Philadelphia, Pennsylvania, United States; Tidal Health, Salisbury, Maryland, United States; University of Pennsylvania, Philadelphia, Pennsylvania, United States; Tidal Health, Salisbury, Maryland, United States; University of Pennsylvania, Philadelphia, Pennsylvania, United States; University of Pennsylvania, Philadelphia, Pennsylvania, United States

## Abstract

A primary reason for visit of abdominal pain in the emergency department (ED) defines a syndromic presentation that can carry morbidity and mortality comparable to a myocardial infarction. Whether imaging decisions in the ED adequately account for this risk, and whether this risk is mediated in part by greater diagnostic complexity remains unknown. In a prospective cohort of 1,169 patient visits to a US community ED, a surgeon codified each visit according to history, examination, laboratory testing, imaging (the acuity of CT findings), diagnosis, and disposition. Chi square tests (two-tailed, alpha=.05) tested for differences between age groups (younger: 18-40, middle: 40-60, older adult: 60+). We found that 42.5% of younger adults received a CT, compared to 66.7% and 70.0% of middle age and older adults (P< 0.001). 20.5% of CT findings were acute in younger, 34.0% in middle, and 39.6% in older adults (P< 0.001). The top 3 acute diagnoses accounted for 75.5% of younger, 62.9% of middle, and 62.7% of older adults, with Herfindahl Indices of 0.25 for younger, 0.16 for middle, and 0.15 for older adults. ED clinicians tested modestly more in older adults, but still potentially over-test younger adults and under-test older adults as judged by test positivity. Older adults had greater diagnostic complexity than younger adults. Dissemination of these findings may improve appropriateness of testing.

